# Impact of oxidative stress on the cytoskeleton of pancreatic epithelial cells

**DOI:** 10.3892/etm.2014.1979

**Published:** 2014-09-18

**Authors:** WEI-GUO HU, QI-PING LU

**Affiliations:** Department of General Surgery, Wuhan General Hospital of Guangzhou Military Command, Wuhan, Hubei 430070, P.R. China

**Keywords:** pancreatic disease, oxidative stress, nuclear factor κ-light-chain-enhancer of activated B cells

## Abstract

In the present study the effect of reactive oxygen species on the morphological changes of pancreatic epithelial cells in a three-dimensional culture system was investigated. In addition, the expression of signaling molecules during this process was determined. Matrigel™ was used to construct a three-dimensional culture model of pancreatic epithelial and cancer cells. The cultured cells were stimulated with 1 or 200 μmol/l H_2_O_2_ (a typical reactive oxygen species), and the morphological changes were then evaluated after 15 min, 1 h and 4 h. The cytoskeleton of the cells was observed using laser scanning confocal microscopy with immunofluorescence staining. In addition, the nuclear content of nuclear factor κ-light-chain-enhancer of activated B cells (NF-κB) was detected using ELISA. The results demonstrated that treatment with 200 μmol/l H_2_O_2_ induced cell contraction after 15 min, and cell morphology recovered after 1 h; however, cell size was reduced after 4 h. Consequently, intracellular actin and microtubules were rapidly lost following H_2_O_2_ treatment, and the cytoskeleton became indistinct and eventually disintegrated after 4 h. Similar observations were noted for the normal pancreatic epithelial and cancer cells. By contrast, treatment with 1 μmol/l H_2_O_2_ did not affect the morphology and cytoskeleton of pancreatic epithelial cells. In addition, 200 μmol/l H_2_O_2_ treatment increased the activity of NF-κB gradually, while 1 μmol/l H_2_O_2_ treatment was found to have little impact on the activity of NF-κB. Therefore, it was demonstrated that oxidative stress can induce the early onset of reversible cell contraction and cytoskeleton depolarization in pancreatic epithelial cells, and can increase NF-κB expression.

## Introduction

Oxidative stress is the cause of numerous diseases. It has previously been shown that oxidative stress has an important role in the pathogenesis of acute pancreatitis (1). Furthermore, it has been demonstrated that chronic inflammation may produce excessive reactive oxygen species and a large number of free radicals, which induce oxidative damage to cells (2).

Growing cell lines in three dimensional culture reduces the complexity of the *in vivo* state and enables the manipulation of culture conditions and functions. The functions of the cultured cells depend on the cytoskeleton, and integrins provide a structural link between extracellular matrix proteins and the actin cytoskeleton. In the classical two-dimensional culture systems, cell culture is a type of plate culture. Cultured cells with extracellular matrix constitute the overall environment; therefore, there are big differences in the biological characteristics from *in vivo* cells. The morphological features are also changed. Application of a three-dimensional culture system provides the basis for the spatial structure and growth of cells. In the present culture system, Matrigel™ was used as the culture medium. A three-dimensional culture system plays an important role in regulating cell growth, differentiation and migration.

In the present study, a three-dimensional model of cultured pancreatic epithelial cells was constructed, and the cells were stimulated with H_2_O_2_. Morphological changes in the pancreatic epithelial cells in the two- and three-dimensional culture systems following H_2_O_2_ treatment were observed. Using the three-dimensional culture model, it was investigated whether pancreatic epithelial cells exhibited cytoskeleton reorganization in response to H_2_O_2_.

## Materials and methods

### Cell culture

The AR42J pancreatic epithelial cell line and Panco2 pancreatic cancer cell line were obtained from the China Center for Type Culture Collection (Wuhan, China). Cells were maintained at 37°C in complete Dulbecco’s minimum essential medium (DMEM) in an atmosphere of 5% CO_2_.

### Three-dimensional cell culture model

Matrigel was used to construct a three-dimensional culture system as follows: 2 ml Matrigel was placed into a culture dish, and 0.3 ml 10X DMEM and 0.25 ml calf serum with AR42J or Panco2 cells were then added to the culture dish, and the cell density was maintained at ~2.5×10^5^. All procedures were performed on ice. The culture dish was placed in an incubator once the gel had formed.

### Study design

H_2_O_2_ was diluted to 1 μmol/l or 200 μmol/l respectively. When cells reached confluence, H_2_O_2_ was added. The cultured cells were incubated with 1 or 200 μmol/l H_2_O_2_ for 15 min, 1 h and 4 h. Stimulated cells were compared with unstimulated controls at each time-point. Following incubation, β-actin and α-tubulin cytoskeletal changes and nuclear factor κ-light-chain-enhancer of activated B cells (NF-κB) expression levels were assessed. The cytoskeletal changes were detected using laser scanning confocal microscopy (LSCM) (LSM 510 META; Carl Zeiss AG, Oberkochen, Germany).

### Fluorescence microscopy

Panco2 and AR42J cells were grown on cover slips. Following fixation, the cells were blocked in phosphate-buffered saline and 0.1% Triton X-100 supplemented with 10% fetal calf serum for 1 h. Monoclonal mouse anti-human antibodies against β-actin (300 μl, 1:250; Santa Cruz Biotechnology, Inc., Santa Cruz, CA, USA) or α-tubulin (300 μl, 1:20; Santa Cruz Biotechnology, Inc.) were added and then incubated in a humid chamber for 16 h. The cells were subsequently observed using LSCM.

### Analysis of NF-κB expression

Cytosolic and nuclear fractions of the AR42J pancreatic epithelial and Panco2 pancreatic cancer cell lines were isolated. The protein content of these cell fractions was analyzed using the Bradford method. The cell fractions were added to a 96-well plate containing a consensus-binding site of oligonucleotides for NF-κB. The NF-κB expression was detected by ELISA, in which NF-κB was captured by a double-stranded oligonucleotide probe containing the consensus-binding sequence for NF-κB. The binding of NF-κB to its consensus sequence was detected using a primary anti-NF-κB antibody (Santa Cruz Biotechnology, Inc.), followed by a secondary antibody conjugated to horseradish peroxidase.

### Statistical analysis

Data were analyzed using SPSS version 12.0 (SPSS, Inc., Chicago, IL, USA). Each treatment group had a sample size of six. Differences between treatment groups were estimated using Dunnett’s multiple range test. P<0.05 was considered to indicate a statistically significant difference.

## Results

### Two-dimensional culture

**Figure 1:**
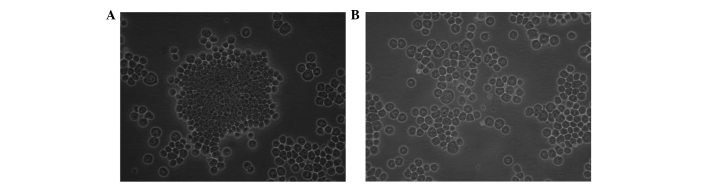
(A) Cells prior to treatment with H_2_O_2_. (B) Cells treated with 200 μmol/l H_2_O_2_ for 15 min. Magnification, ×200.

The morphological changes in the normal AR42J pancreatic epithelial cells cultured in the two-dimensional system and treated with 200 μmol/l H_2_O_2_ for 15 min were observed. It was found that cells grew to confluent monolayers; however, following H_2_O_2_ treatment the cells were dispersed and reduced in size (Fig. 1).

**Figure 2:**
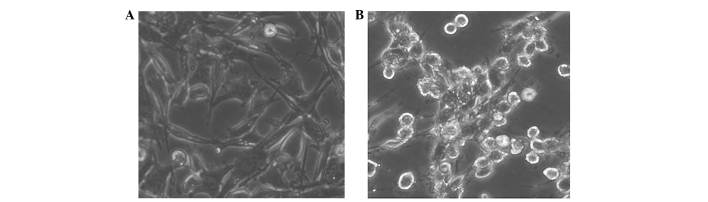
(A) Cells prior to treatment with H_2_O_2_. (B) Cells treated with 200 μmol/l H_2_O_2_ for 15 min. Magnification, ×400.

In addition, the cytoskeletal changes in the normal AR42J pancreatic epithelial cells cultured in the two-dimensional system and treated with H_2_O_2_ were analyzed. It was observed that H_2_O_2_ induced the rearrangement of the cytoskeleton. Following 15 min incubation with 200 μmol/l H_2_O_2_, the cultured cells contracted, and cell atypia was observed in all cultures after 1 h. However, 1 μmol/l H_2_O_2_ did not induce cell atypia (Fig. 2).

### Three-dimensional culture

Cytoskeletal changes in the pancreatic epithelial cells cultured in the three-dimensional system were observed. In the untreated cells, the actin cytoskeleton (green) appeared as a dense network of irregular filaments randomly oriented through the cytoplasm. Tubulin filaments (red) appeared thinner and better defined compared with actin, running primarily along the cellular long axis. Cells treated with 1 μmol/l H_2_O_2_ did not differ in morphology from those in the control cultures, regardless of incubation time. Similar observations were noted for the normal pancreatic epithelial and cancer cells.

**Figure 3:**
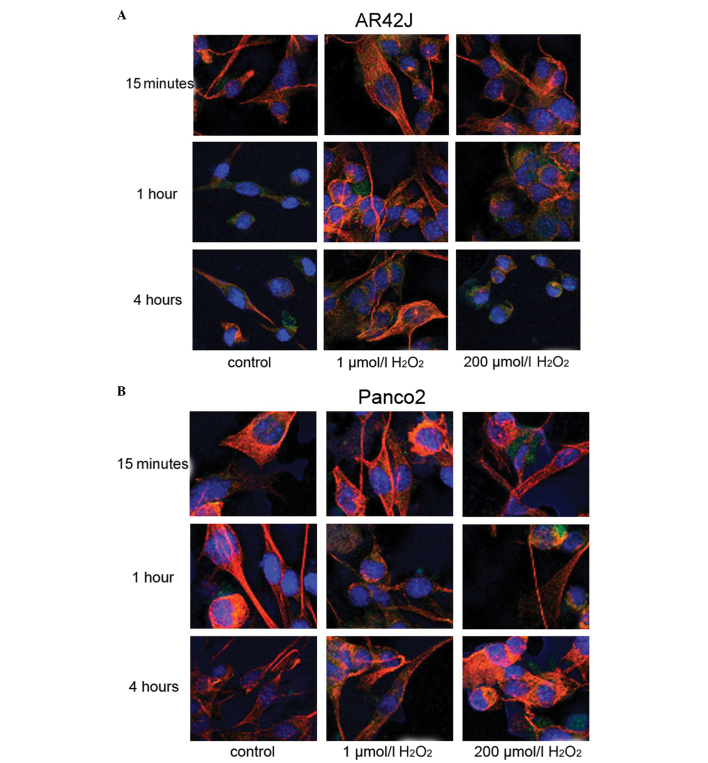
Cytoskeletal changes in (A) AR42J and (B) Panco2 cells (magnification, ×400).

When the cells were treated with 200 μmol/l H_2_O_2_ for 15 min, the actin cytoskeleton showed significant reorganization, as shown in Fig. 3. Additionally, the filamentous structure of actin and tubulin disappeared in these cells. When the cells were treated for 1 h, the actin and tubulin filaments normalized a little. Four hours after H_2_O_2_ treatment, the cell cytoskeleton structure became thicker and coarser than that in the control group. However, 1 μmol/l H_2_O_2_ treatment had no marked effect on the cytoskeleton.

### NF-κB expression

**Figure 4:**
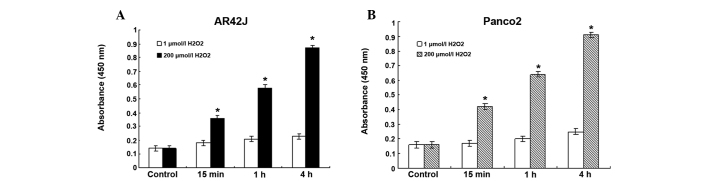
Nuclear factor κ-light-chain-enhancer of activated B cells activation in (A) AR42J and (B) Panco2 cells (^*^P<0.05 vs. control cells with the same concentration H_2_O_2_ treatment).

As shown in Fig. 4, when the cells were treated with 1 μmol/l H_2_O_2_, the expression level of NF-κB did not change significantly. However, treatment with 200 μmol/l H_2_O_2_ significantly increased the level of NF-κB in pancreatic epithelial cells (P<0.05). When the cells were treated with 200 μmol/l H_2_O_2_, the expression of NF-κB began to increase 15 min after treatment. After 4 h, the expression level of NF-κB was almost two-fold that of the level at the 15-min point in the same group.

## Discussion

Oxidative stress is the cause of numerous diseases. It is caused directly or indirectly by the generation of reactive oxygen species in the body and the imbalance between the pro- and antioxidants (3). It has been shown that chronic inflammation may produce excessive reactive oxygen radicals, which induce oxidative damage to cells (4). Chronic pancreatitis (CP) is a common disease worldwide. However, the pathogenesis of this disease is not clear. At present, there is not a specific diagnostic method and effective treatment for CP. Therefore, the pathogenesis of CP, and its clinical and pathological diagnosis are of particular importance.

Oxidative stress may cause epithelial cell dysfunction or death (5). H_2_O_2_ has been found to affect epithelial cell function by activating certain redox-sensitive transcription factors (6) or protein kinase C translocation (7). Low doses of H_2_O_2_ can induce actin rearrangement. However, the effects of H_2_O_2_ on other components of the cytoskeleton have yet to be elucidated (8). In the present study, the effect of different exposure times and concentrations of H_2_O_2_ on the cytoskeleton and morphology of pancreatic epithelial cells was investigated. Cultured AR42J pancreatic epithelial and Panco2 pancreatic cancer cells were fixed at different time-points (15 min, 1 h and 4 h) after stimulation with H_2_O_2_, and stained with anti-tubulin and anti-actin antibodies.

Pancreatic carcinoma is characterized by its aggressive local invasion of adjacent structures. At the time of first diagnosis, only 10–20% of cases are eligible for the potentially curative Whipple’s procedure. Furthermore, pancreatic cancer is relatively resistant to chemotherapy and radiotherapy. Therefore, an enhanced understanding of the biological role of the genotypic changes that occur during pancreatic carcinogenesis may provide novel ideas for the development of strategies to prevent and treat this disease. It is already known that persistent inflammation may favor the malignant transformation of pancreatic ductal cells, leading to dysplasia and, ultimately, cancer. In patients with CP, the acinar injury may recur and lead to repeated inflammation with the continuous infiltration of inflammatory cells, eventually leading to atrophy and fibrosis (9). The risk of developing pancreatic cancer in patients with hereditary pancreatitis is 53-fold that of the risk in unaffected individuals, which is higher than the risk noted with numerous other inflammatory diseases (10). Therefore, the aim of the present study was to determine the impact of oxidative stress on pancreatic epithelial and pancreatic cancer cells, and to investigate the association between oxidative stress and pancreatic cancer.

The establishment and maintenance of epithelial cell polarity is important for the normal function of certain organs (11). The formation and maintenance of cell polarity depends on cell-cell and cell-matrix contacts, such as junction complexes, focal adhesions and the cytoskeleton (12). The cytoskeleton is important for cell polarity. A number of cytoskeletal factors affect cell shape and polarity.

In the classical two-dimensional culture system, cells are grown in a flask with a flat bottom. The extracellular matrix is different from that of the *in vivo* environment, which results in different biological characteristics. Therefore, this type of culture method does not reflect the *in vivo* status. The biological and morphological characteristics of these cultured cells are changed. The three-dimensional culture system uses Matrigel to constitute the three-dimensional extracellular matrix. This extracellular matrix provides space and mechanical structure for cell growth, and has an important role in regulating cell migration and differentiation (13).

In the present study, Matrigel was used to construct a three-dimensional culture model of pancreatic epithelial cells. H_2_O_2_ was used to mimic *in vivo* oxidative stress. The effect of reactive oxygen species on the cytoskeletal and morphological changes in pancreatic epithelial cells cultured in the three-dimensional system were observed. The results demonstrated that oxidative stress induces the early onset of reversible cell contraction and cytoskeleton depolarization in pancreatic epithelial cells, together with increased activity of NF-κB.

The cytoskeleton consists of microfilaments, microtubules and intermediate filaments, all of which are important in the maintenance of cell shape. Changes in these filaments allow rapid changes in the cell three-dimensional structure (14). Microfilaments are the contractile protein actin in monomeric and filamentous form (15). Microtubules are essential for cell division and intracellular transport. They are composed of tubulin subunits together with ancillary microtubule-associated proteins (16). NF-κB is a ubiquitous transcriptional factor, which regulates the expression of cytokines, growth factors and cell adhesion molecules. NF-κB can be activated by cytokines, free radicals, inhaled particles, ultraviolet radiation and the products of certain bacteria and viruses. The abnormal activation of NF-κB is correlated with a number of autoimmune inflammatory diseases, including arthritis, pulmonary fibrosis and tumors (17). In the present study it was found that oxidative stress in pancreatic epithelial cells increased the expression of NF-κB, and the expression levels were consistent with the changes in the cytoskeleton, suggesting that there is a link between NF-κB and the cytoskeleton.

In an experimental pancreatic cancer model, N-nitrosobis(2-oxopropyl)amine has been shown to induce an increase in lipoperoxides and a reduction in antioxidants in the pancreas. This indicates that there is a close association between oxidative stress and pancreatic cancer (18). The present study suggests that reactive oxygen species may induce the destruction of the cytoskeleton in epithelial cells, followed by the activation of NF-κB, which is important for the progression of pancreatic cancer. These results may provide evidence to clarify the association between oxidative stress and pancreatic cancer.
